# Retraction: MiR-182-5p and miR-96-5p increased hepatocellular carcinoma cell mobility, proliferation and cisplatin resistance partially by targeting RND3

**DOI:** 10.1039/d1ra90019f

**Published:** 2021-01-20

**Authors:** Laura Fisher

**Affiliations:** Royal Society of Chemistry Thomas Graham House, Science Park, Milton Road Cambridge CB4 0WF UK advances-rsc@rsc.org

## Abstract

Retraction of ‘MiR-182-5p and miR-96-5p increased hepatocellular carcinoma cell mobility, proliferation and cisplatin resistance partially by targeting RND3’ by Shiming Yang *et al.*, *RSC Adv.*, 2018, **8**, 34973–34983, DOI: 10.1039/C8RA07055E.

The Royal Society of Chemistry hereby wholly retracts this *RSC Advances* article due to concerns with the reliability of the data. The images in the article were screened by an image integrity expert who raised concerns with the integrity of the western blot panels and identified instances of image duplication.

All the western blot panels have no visible background, but do not appear to be over-contrasted. The individual bands are uncharacteristically very uniform and regular in shape, indicating that they may not be genuine.

In Fig. 5E, parts of the images in panels 1 and 5 are duplicated in panel 4.

The authors were asked to provide the raw data for this article, but did not respond. Given the significance of the concerns about the validity of the data, and the lack of raw data, the findings presented in this paper are not reliable.

The authors have been informed but have not responded to any correspondence regarding the retraction.

Signed: Laura Fisher, Executive Editor, *RSC Advances*

Date: 7^th^ January 2021

## Supplementary Material

